# Total hip arthroplasty for fractured neck of femur does not restore preoperative hip-specific function, health-related quality of life, or level of fitness

**DOI:** 10.1007/s00590-024-04034-1

**Published:** 2024-06-26

**Authors:** Lucas Ho, Benjamin Ford, Paul Gaston, Nick D. Clement

**Affiliations:** 1 Edinburgh Medical School, University of Edinburgh, Chancellor’s Building, Little France, Edinburgh, EH16 4SB UK; 2 Edinburgh Orthopaedics, Royal Infirmary of Edinburgh, Little France, Edinburgh, UK; 3 Department of Orthopaedics and Usher Institute, University of Edinburgh, Little France, Edinburgh, UK

**Keywords:** Hip, Fracture, Function, HRQoL, Fitness, Total hip arthroplasty

## Abstract

**Purpose:**

The primary aim was to assess whether a total hip arthroplasty (THA) was able to restore health-related quality of life (HRQoL) following an intracapsular hip fracture. The secondary aims were to assess changes in hip-specific function, fitness/frailty, mortality risk, complications and revision risk, and factors independently associated with these.

**Methods:**

This retrospective cohort study included all patients aged ≥ 50 years admitted with a hip fracture from the emergency department at a single centre during a 42-month period. Patient demographics, perioperative variables, complications, revision, and mortality were collected. Patient-reported outcome measures (PROMs) were assessed at final follow-up.

**Results:**

Among 250 identified patients, 189 (75.6%) were women with a mean age of 70.3 (range 50–94 years). Mean follow-up was 2.3 (SD 1.1) years. The implant and patient survival rates at 2 years were both 95.5% (95% confidence intervals (CI) +/− 2.7). Older age (hazard ratio [HR] 1.22, 95% CI 1.12–1.33, *p* < 0.001) and male sex (HR 3.33, 95% CI 1.15–10.0, *p* = 0.026) were independently associated with mortality. There were 19 (7.6%) postoperative complications that included 6 (2.4%) periprosthetic fractures, 5 (2.0%) deep infections, and 8 (3.2%) dislocations, of which 13 underwent revision. Increasing time to theatre (HR 1.02, 95% CI 1.01–1.03, *p* = 0.017) was independently associated with a postoperative complication. Postoperative PROMs were available for 166 (66.4%) patients. There were significant (*p* < 0.001) deteriorations in EuroQol-5D (Mean difference [MD] 0.192, 95% CI 0.133–0.252), Oxford hip score (MD 2.5, 95% CI 1.5–3.6), and fitness (Rockwood score MD 0.7, 95% CI 0.5–0.8) relative to preoperative levels of function.

**Conclusion:**

THA may be the treatment of choice in a physically active patient with the aim of restoring their HRQoL, hip function, and fitness, but this was not observed. Furthermore, there was a high complication rate which was associated with increasing time to theatre.

**Level of evidence:**

III, retrospective cohort study.

## Introduction

Intracapsular hip fractures are common in the elderly population and are associated with a significant burden of morbidity and mortality [1, 2]. Each year in the UK, there are over 65,000 reported cases of hip fractures, with approximately half of them presenting as intracapsular hip fractures [3]. By 2029, it is estimated that there will be a 32% increase in individuals presenting with a hip fracture to healthcare services, with older adults experiencing the biggest relative increase in volume over time. Based on these projections, the overall length of hospital stay following hip fracture will increase by 60,699 days per year, incurring an additional cost of approximately £25 million [4]. Given this demographic shift towards an ageing and increasingly multimorbid population, it is imperative to understand the outcomes associated with hip arthroplasty for intracapsular hip fractures.

The aim of a THA for an intracapsular hip fracture is to restore a patient’s HRQoL to the fullest extent possible. The current guidelines from the National Institute of Health and Care Excellence (NICE) recommend total hip arthroplasty (THA) over hemiarthroplasty (HA) for patients capable of mobilising independently with minimal aid, are cognitively intact, and medically fit for the procedure [5]. This guidance is founded on the rationale that THA generally results in superior functional outcomes compared to HA [6, 7] as fitter patients are more likely to tolerate a bigger operation and benefit from better functional outcomes. There is however conflicting evidence as to whether THA provides a clinically significant improvement over HA in functional outcomes and quality of life [5, 8]. It is also well documented that THA involves longer operation duration, increased blood loss, and higher rates of dislocation and revision surgery compared to HA [9–11]. There is less evidence regarding the incidence and risk of periprosthetic fractures specifically in patients undergoing THA for intracapsular hip fractures. Furthermore, there is a lack of comprehensive assessments pertaining to changes in frailty/fitness and hip-specific function following THA, and more specifically, the authors are not aware of any studies investigating the restoration of patients to their pre-injury functional status following THA. This study aims to address gaps in the knowledge of the functional outcome following THA for a hip fracture, thereby enhancing the ability to inform patients about potential, functional outcomes, risks and benefits associated with THA. Additionally, it will assist clinicians in optimising their practices to prevent and minimise adverse outcomes in patients undergoing hip arthroplasty procedures. The primary aim of this study was to assess whether a THA was able to restore HRQoL following an intracapsular hip fracture. The null hypothesis was that there was no difference in HRQoL following THA. Secondary aims were to assess changes in hip-specific function, fitness/frailty, mortality, complication, and revision risks, and identify factors independently associated with these outcomes.

## Materials and methods

This retrospective cohort study involved all patients aged 50 and above who were admitted with acute hip fractures to a large orthopaedic trauma centre over a 42-month period (1st January 2019–30th June 2022). Serving a population of about 850,000, this trauma centre manages approximately 1300 hip fractures annually [32]. The inclusion criteria were patients with intracapsular hip fractures who lived within the catchment area and underwent THA. Exclusion criteria were isolated fractures of the acetabulum, pubic ramus, greater trochanter, and periprosthetic fractures. Patients were retrospectively identified from the local hip fracture database, with continuous prospective data collection as part of the national Scottish Hip Fracture Audit (SHFA) [32]. Data on patient demographics, fracture type, time to theatre, ASA grade, length of stay, and mortality were collected from electronic health records (EHRs) (TrakCare, InterSystems Corporation, MA, USA) and contemporaneous documentation. Time to theatre was measured according to SHFA guidelines, from ward admission to the start of anaesthesia. Specialist local audit coordinators, familiar with hip fractures and the trauma unit, compiled the data, which were then reviewed for completeness as part of SHFA’s routine activities. All data handling complied with the UK Caldicott principles. Ethical approval for data collection/analysis was obtained from the Scotland Research Ethics Committee (Ref No 20/SS/0125).

Each patient’s socioeconomic status was determined using the Scottish Index of Multiple Deprivation (SIMD), which evaluates seven domains: current income, employment, health, education, skills and training, housing, geographic access, and crime [12]. In this study, the most recent SIMD rankings published in 2020 were employed to categorise patients into quintiles of local data zone deprivations, ranging from 1 (most deprived) to 5 (least deprived), based on their postcode at the time of injury.

### Outcomes

Patient mortality status was sourced from the EHR of the local hospital, the sole healthcare provider for the catchment population. This information was retrieved using each patient’s Community Health Index (CHI) number, a unique national patient identifier. Patient-reported outcome measures (PROMs) were gathered postoperatively, encompassing inquiries into both pre and postoperative outcomes.

The Oxford hip score (OHS) is a patient-reported outcome measure designed to assess the effects of pain and functional limitations in individuals undergoing hip arthroplasty [13]. Comprising 12 questions, it employs a 5-item Likert response format and yields scores ranging from 0 to 48, with higher scores indicating improved outcomes. Widely validated and commonly used in THA patients, the OHS has a minimal clinically important difference (MCID) of 8 points [14].

The EuroQol-5D (EQ-5D) questionnaire assesses general health across five domains: mobility, self-care, usual activities, pain/discomfort, and anxiety/depression [15]. Utilising the three-level version (3L) of the EQ-5D questionnaire, responses for each domain are recorded at three severity levels (none/slight problems; moderate/severe; or unable/extreme problems) [16]. Permission to utilise the UK interviewer-administered version of the EQ-5D-3L was obtained from the EuroQol Research Foundation (Marten Meesweg 107, 3068 AV Rotterdam, Netherland). The index ranges from − 0.594 to 1, with a score of 1 representing perfect health and 0 representing death. Scores below zero on the EQ-5D represent a health state considered worse than death [17]. The MCID for the EQ-5D score post-THA is 0.08; therefore, a change of 0.08 or more in the score was deemed as clinically significant [18].

The Rockwood Clinical Frailty Scale (CFS) was used to determine the level of frailty [19]. This scale rates patients on a scale of 1–9, with 1 indicating “very fit” and 9 indicating “terminally ill”. Frailty status was assessed both at the time of assessment and 6 months prior to assessment. The CFS is validated for retrospective application [20].

### Data analyses

Data analyses were performed using Statistical Package for the Social Sciences (SPSS) software (IBM, Inc., Armonk, New York, USA) version 17. Independent (age, time to theatre) and paired (PROMs) Student’s *t*-tests were used to assess significant differences in continuous variables between groups and changes from preoperative to postoperative states. Categorical variables (sex, SIMD, and ASA grade) were assessed using a Chi-squared test for between-group comparisons. Kaplan–Meier time-to-event methodology was used to assess patient survival. Cox regression analysis was used to assess the independent association of factors with patient mortality when adjusting for confounding variables. The predictive value of age on mortality was evaluated using a receiver operative characteristic (ROC) curve, with the area under the curve (AUC) ranging from 50% (no predictive value) to 100% (perfect predictor). Statistical significance was defined as a *p*-value of < 0.05.

A power calculation was performed, based on the MCID for the EQ-5D score as a measure of HRQoL which is 0.08 and has a standard of 0.3 [21]. To achieve 90% power and using an alpha of 0.05 (two tailed), a minimum of 147 patients would be required. Therefore, to achieve the number of patients required to meet the power calculation with an estimated 3-year mortality of 10% and loss to follow-up of 30% [22], a 42-month study period was chosen.

## Results

During the study period, 250 patients presented to the ED with an acute hip fracture that had a THA for an intracapsular hip fracture. There were 189 (75.6%) women with a mean age of 70.3 (range 50–94 years). Mean time to theatre, from admission to the ward, was 26.2 (SD 17.3) hours. Median length of acute hospital stay was 6 (interquartile range 4–8) days. The mean follow-up was 2.3 (SD 1.1) years.

**Fig. 1:**
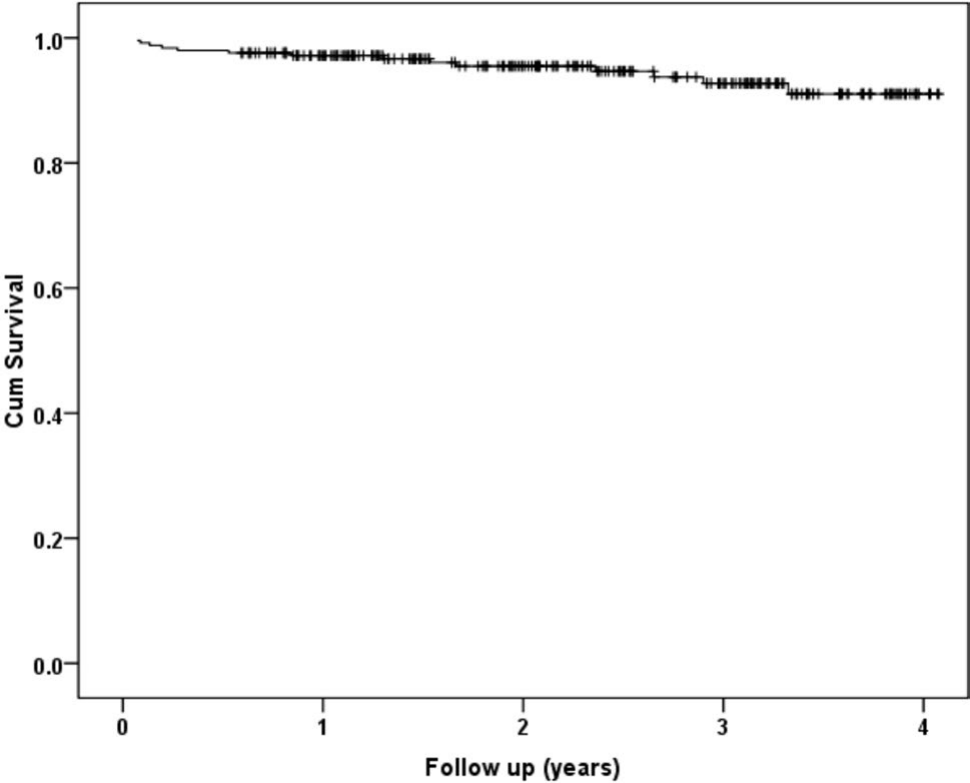
Kaplan–Meier curve for patient survival following total hip arthroplasty for a hip fracture

**Fig. 2:**
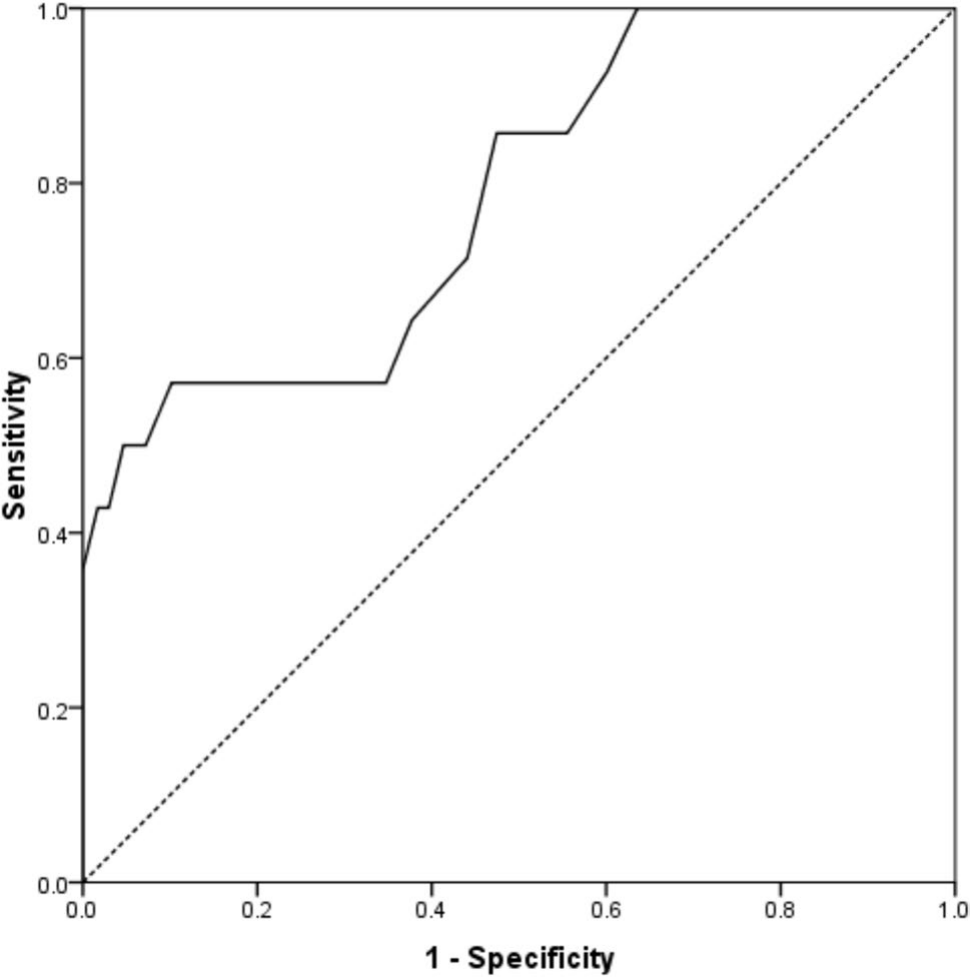
Receiver operator characteristic curve for age as a predictor for mortality following total hip arthroplasty for a hip fracture

During the follow-up period, there were 14 (5.5%) deaths identified. The survival rates according to follow-up are shown in Table 1 and Fig. 1. Male sex (*p* = 0.022), older age (*p* < 0.001), and greater ASA grade (*p* < 0.001) were associated with mortality following THA (Table 2). When adjusting for confounding factors, older age (hazard ratio (HR) 1.22, 95% confidence intervals (CI) 1.12–1.33, *p* < 0.001) and male sex (HR 3.33, 95% CI 1.15–10.0, *p* = 0.026) were independently associated with mortality. Older age was a moderate predictor of mortality, with an AUC of 78.5% (95% CI 65.7–91.2, *p* < 0.001) and a threshold of 74 years was associated with 64.3% sensitivity and 65.3% specificity (Fig. 2).

**Table 1:** Patient (death as endpoint) and implant (revision as endpoint) survival following a total hip arthroplasty for a hip fracture at different timepoints

Survival	30 days	90 days	1 year	2 years	3 years
Patient	99.6 (± 0.8)	98.4 (± 1.4)	97.2 (± 2.2)	95.5 (± 2.7)	92.7 (± 4.1)
Implant	98.8 (± 1.4)	97.6 (± 2.0)	97.2 (± 2.0)	95.5 (± 2.7)	92.9 (± 3.9)

**Table 2:** Patient demographics, SIMD, ASA grade, and time to theatre according to mortality following hip fracture for the study cohort

Demographic	Descriptive	Status		Difference/odds ratio (95% CI)	*p*-value
		Alive	Dead		
Sex *n* (%)	Male	54 (21.6)	7 (2.8)	Odds Ratio 0.30 (0.10 to 0.88)	0.022*
	Female	182 (72.8)	7 (2.8)		
Age (years: mean, SD)		69.7 (8.3)	80.3 (8.9)	Difference 10.6 (6.0 to15.1)	< 0.001**
SIMD *n* (%)	1 (Most deprived)	16 (6.4)	1 (0.4)		0.819*
	2	50 (20.0)	4 (1.6)		
	3	44 (17.6)	2 (0.8)		
	4	47 (18.8)	4 (1.6)		
	5 (Least)	79 (31.6)	3 (1.2)		
ASA Grade *n* (%)	1	29 (11.6)	0 (0)		< 0.001*
	2	175 (70.0)	5 (2.0)		
	3	32 (12.8)	9 (3.6)		
	4	0 (0)	0 (0)		
Time to theatre (hours: mean, SD)		29.4 (17.0)	35.6 (22.5)	Difference 6.2 (− 3.2 to 15.6)	0.193**
Revised *n* (%)	No	225 (90.0)	12 (4.8)	Odds Ratio 3.41 (0.7 to 17.1)	0.115
	Yes	11 (4.4)	2 (0.8)		

*SIMD* Scottish Index of Multiple Deprivation, *ASA* American Society of Anaesthesiology

*Chi-square test, **Student’s *t*-test

**Fig. 3:**
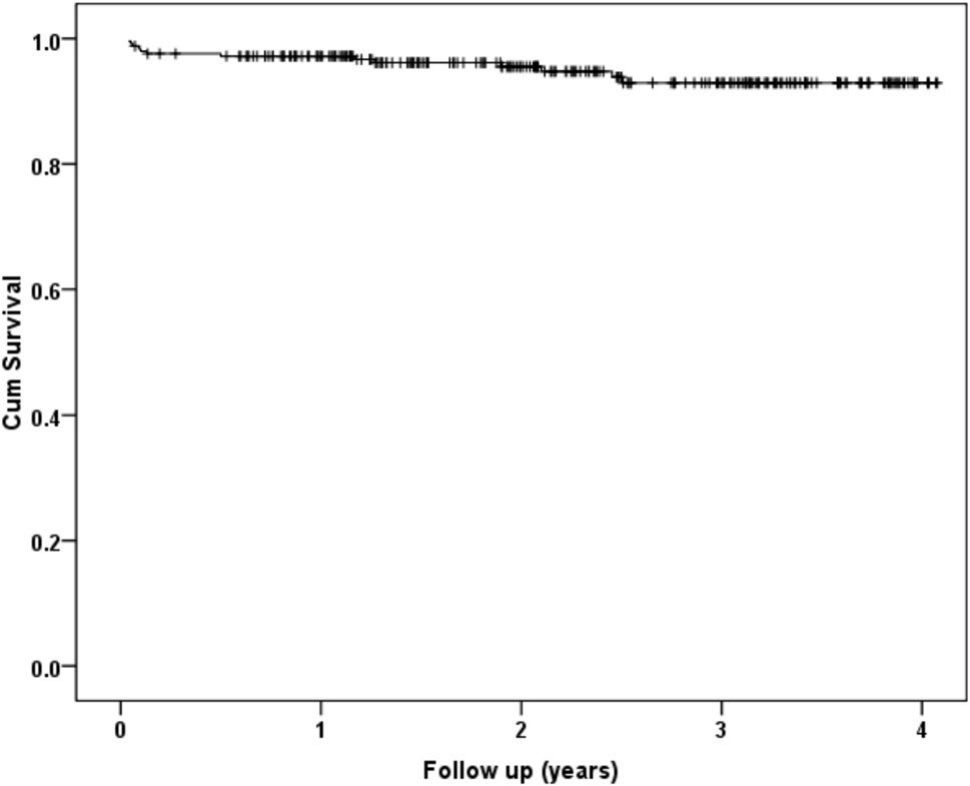
Kaplan–Meier curve for implant survival (revision as endpoint) following total hip arthroplasty for a hip fracture

There were 19 (7.6%) postoperative complications of which there were 6 (2.4%) periprosthetic fractures, 5 (2.0%) deep infections, and 8 (3.2%) dislocations. One patient had a dislocation and then had a subsequent deep infection requiring debridement, antibiotics, and implant retention; therefore, 18 patients had a serious complication. Increasing time to theatre (*p* = 0.006) was associated with postoperative complications (Table 3). When adjusting for confounding factors, increasing time to theatre (HR 1.02 per hour, 95% CI 1.01–1.03, *p* = 0.017) was independently associated with a postoperative complication. Of the eight dislocations, five underwent revision, and of the six periprosthetic fractures, four underwent revision (three ORIF and one revision) for B2-type fractures, and the other two were type A that were managed conservatively. Therefore, 13 revisions were undertaken in the study cohort and survival is shown in Table 1 and Fig. 3. When adjusting for confounding factors, increasing time to theatre (HR 1.02 per hour, 95% CI 1.01–1.04, *p* = 0.010) was independently associated with a postoperative revision.

**Table 3:** Patient demographics, SIMD, ASA grade, time to theatre, frailty, and functional measures according to complication status following a total hip arthroplasty for a hip fracture

Demographic	Descriptive	Complication		Difference/odds ratio (95% CI)	*p*-value
		Yes	No		
Sex *n* (%)	Male	4 (1.6)	57 (22.8)	Odds Ratio 1.14 (0.36 to 3.6)	0.823*
	Female	14 (5.6)	175 (70.0)		
Age (years: mean, SD)		73.0 (11.7)	70.1 (8.3)	Difference 2.9 (−1.3 to 7.1)	0.173**
SIMD *n* (%)	1 (Most deprived)	1 (0.4)	16 (6.4)	N/A	0.622*
	2	2 (0.8)	52 (20.8)		
	3	5 (2.0)	41 (16.4)		
	4	5 (2.0)	46 (18.4)		
	5 (Least)	5 (2.0)	77 (30.8)		
ASA Grade *n* (%)	1	27 (10.8)	2 (0.8)	N/A	0.391*
	2	168 (67.2)	12 (4.8)		
	3	37 (14.8)	4 (1.6)		
	4	0 (0)	0 (0)		
Time to theatre (hours: mean, SD)		40.6 (32.0)	28.9 (15.5)	Difference 11.6 (3.4 to 19.9)	0.006**
Rockwood frailty score		2.4 (0.8)	2.6 (1.2)	Difference 0.2 (− 0.5 to 0.9)	0.642**
Oxford Hip Score (mean, SD)		44.3 (5.4)	45.7 (5.8)	Difference 1.4 (− 2.3 to 5.1)	0.445**
EQ-5D (mean, SD)		0.817 (0.220)	0.823 (0.318)	Difference 0.006 (− 0.196 to 0.208)	0.951**

*SIMD* Scottish Index of Multiple Deprivation, *ASA* American Society of Anaesthesiology

*Chi-square test, **Student’s t-test

Postoperative PROMs were available for 166 (70.3%) patients of the 236 that were alive at a follow-up of 2.2 years (SD 1.0). Of the 70 patients lost to follow-up to PROMs, 47 were uncontactable and 23 refused to participate. There were no differences in sex (*p* = 0.982), age (*p* = 0.09), SIMD *p* = 0.820), and ASA grade (*p* = 0.627) between those lost to follow-up and those responding to PROMs. There were significant (*p* < 0.001) deteriorations in hip-specific function (OHS) and HRQoL (EQ-5D) relative to preoperative levels of function (Table 4). This was also associated with an increased Rockwood frailty score (Table 4).

**Table 4:** Preoperative and postoperative Oxford hip scores, EQ-5D, and Rockwood frailty scores and the changes relative to preoperative scores for the study cohort (*n* = 166)

PROM and timepoint	Group				Difference (95% CI)	*p*-value*
	Pre-THA		Post-THA			
	Mean	SD	Mean	SD		
Oxford Hip Score	45.6	5.7	43.1	7.3	2.5 (1.5 to 3.6)	< 0.001
EQ-5D	0.822	0.313	0.630	0.406	0.192 (0.133 to 0.252)	< 0.001
Rockwood Frailty	2.6	1.2	3.3	1.5	0.7 (0.5 to 0.8)	< 0.001

*PROM* patient-reported outcome measure, *THA* Total hip arthroplasty *Paired Student’s *t*-test

## Discussion

This retrospective cohort study aimed to evaluate the impact of THA on restoring HRQoL following intracapsular hip fracture, evaluate changes in hip-specific function, fitness/frailty, mortality, complication and revision risks, and identify factors independently associated with these outcomes. There was a significant decline in hip-specific function (OHS) and HRQoL (EQ-5D), along with an increase (worsening) in patient frailty relative to pre-injury status. After adjusting for confounding factors, older age and male sex were independently associated with increased mortality risk postoperatively. There were 19 (7.6%) postoperative complications, and increasing time to theatre was independently associated with both postoperative complications and revision.

Limitations of the study include a single-centre retrospective design which involves a relatively smaller patient group compared to registry-based analyses. However, registry-based analyses often lack the level of detail that is available in smaller datasets, which allowed adequate adjustment for confounding factors. Another limitation pertains to the use of PROMs, which are subjective evaluations susceptible to external influences, such as memory issues [23]. Older age, often associated with memory problems and cognitive decline [24], may impact the accuracy of recalling pre-injury functionality. Studies report that up to 47% of elderly individuals with hip fractures may have underlying cognitive impairment [25], potentially introducing a misrepresentation of pre-injury status and compromising the reliability of preoperative and postoperative comparisons. Furthermore, unrealistic expectations or a lack of understanding regarding the limitations of the intervention may contribute to perceived dissatisfaction with postoperative outcomes.

During the follow-up, 14 patient deaths occurred. Both male sex and older age were independently associated with increased mortality after adjusting for confounding factors, a finding consistent with studies by Comba et al. [26] and Memtsoudis et al. [27]. The exact reasons for the increased mortality in men following lower limb arthroplasty remain unclear, although hormonal differences between sexes have been proposed as a possible protective factor for female patients [26]. Given that both male sex and increasing age are non-reversible and non-adjustable risk factors, it raises the question of whether gender and age should be considered when deciding the best treatment option for intracapsular hip fracture. Despite the significantly higher mortality in men, the majority of hip fracture patients are women, with incidence rates increasing with age [28]. However, this does not diminish the importance of a management option with reduced mortality in men sustaining intracapsular hip fractures, particularly considering the projected surge in hip fracture incidence by 2029 [4].

Previous studies have identified that time to theatre is a potential reversible risk factor associated with increased mortality following hip fracture fixation [29], although this was not observed in the current study. This discrepancy is likely due to the relatively small sample size of patients in this current study (*n* = 250). This is however consistent with the findings of Farrow et al. [30] which similarly found no association of time to theatre with mortality risk in those undergoing THA for a hip fracture based on national registry data. However, the findings of the current study found that increased time to theatre was independently associated with an increased risk of postoperative complications and revision surgery, after adjusting for confounding factors. Factors contributing to increased time to theatre include limited theatre availability, lack of theatre staff, and stabilisation of the patient’s medical conditions [31]. A previous study demonstrated that implementing the “Big 6”, a series of interventions aimed at optimising patients with hip fractures for early surgery in the emergency department, resulted in a 2-h shorter time to theatre, and thus a reduction in patient mortality following acute hip fracture repair, although this was not specific to patients receiving THA [32]. Common complications leading to THA revision, such as dislocation, periprosthetic fracture, and infection [33], were observed in our patient group. In this study, the incidence rate of postoperative complications was 7.6%. Previous studies have consistently identified advancing age, rheumatoid arthritis, obesity, and ASA > 2 as factors associated with increased risk of postoperative complications [34–36]. Revision surgery is widely recognised for its association with longer operating times, intraoperative risk, increased complication rates and patient morbidity, and inferior outcomes compared to primary THA [37, 38]. The increased incidence of revisions not only leads to diminished theatre availability but also contributes to prolonged hospital stays. Targeted strategies to optimise preoperative risk assessment and postoperative care based on identified risk factors should be implemented to mitigate potential postoperative complications and revision surgeries.

The current NICE guidelines recommend THA over HA for patients capable of mobilising independently with minimal aid, are cognitively intact, and medically fit for the procedure [5]. The prevailing belief is that THA generally yields superior functional outcomes compared to HA [6, 7]. The current study shows a significant reduction in hip-specific function (OHS) and HRQoL (EQ-5D), along with an increase in patient frailty relative to pre-injury status. To the knowledge of the authors, there is currently no existing literature that explores the restoration of patients to their pre-injury status following a THA for an intracapsular fracture. Therefore, although THA may be thought to offer a better functional outcome relative to HA [6, 7], patients should be made aware of their potential deterioration in function following their injury.

These findings may be attributed to individual factors such as age, overall health, and comorbidities, which can impact an individual’s ability to fully regain preoperative function [39, 40]. The mean age of patients in this study is 70.3, a decade younger than the average of those who sustain a hip fracture [32]. Elderly individuals are more susceptible to postoperative cognitive dysfunction [24] and exhibit lower postoperative activity levels compared to younger patients [41] posing challenges in rehabilitation and recovery. Lack of engagement in or adherence to a comprehensive rehabilitation programme may hinder the achievement of optimal functional outcomes. Moreover, older patients often have pre-existing comorbidities, which can further complicate their recovery process and negatively impact overall functional outcomes. A comparable study by Clement et al. [41] reported that increasing age did not influence hip-specific functional outcome or HRQoL following THA; however, their patient cohort comprised individuals undergoing THA for degenerative joint disease. Fractured neck of femur, especially intracapsular fractures, inflicts significant trauma and damage to the hip joint. The severity and complex nature of these fractures pose challenges to the complete restoration of preoperative function. Furthermore, while THA addresses the patient’s fracture, it does not fully replicate the native anatomy and biomechanics of the hip joint [42] which could result in limitations concerning range of motion, stability, and overall function.

## Conclusion

THA may be the treatment of choice in physically active patients with the aim of restoring their HRQoL, hip function, and fitness, but this was not observed. Older age and male sex were independently associated with increased mortality risk postoperatively. There was a high complication rate that affected approximately one in 12 patients which was associated with increasing time to theatre.
